# Opening the Bandgap of Metallic Half‐Heuslers via the Introduction of d–d Orbital Interactions

**DOI:** 10.1002/advs.202302086

**Published:** 2023-06-04

**Authors:** Airan Li, Madison K. Brod, Yuechu Wang, Kejun Hu, Pengfei Nan, Shen Han, Ziheng Gao, Xinbing Zhao, Binghui Ge, Chenguang Fu, Shashwat Anand, G. Jeffrey Snyder, Tiejun Zhu

**Affiliations:** ^1^ State Key Laboratory of Silicon Materials School of Materials Science and Engineering Zhejiang University Hangzhou 310058 China; ^2^ Department of Materials Science and Engineering Northwestern University Evanston IL 60208 USA; ^3^ Information Materials and Intelligent Sensing Laboratory of Anhui Province, Key Laboratory of Structure and Functional Regulation of Hybrid Materials of Ministry of Education, Institutes of Physical Science and Information Technology Anhui University Hefei 230601 China; ^4^ Materials Sciences Division Lawrence Berkeley National Laboratory Berkeley CA 94720 USA

**Keywords:** bandgap engineering, d–d orbital interactions, electronic structure, half‐Heusler, semiconductors

## Abstract

Half‐Heusler compounds with semiconducting behavior have been developed as high‐performance thermoelectric materials for power generation. Many half‐Heusler compounds also exhibit metallic behavior without a bandgap and thus inferior thermoelectric performance. Here, taking metallic half‐Heusler MgNiSb as an example, a bandgap opening strategy is proposed by introducing the d–d orbital interactions, which enables the opening of the bandgap and the improvement of the thermoelectric performance. The width of the bandgap can be engineered by tuning the strength of the d–d orbital interactions. The conduction type and the carrier density can also be modulated in the Mg_1‐_ *_x_* Ti*_x_* NiSb system. Both improved n‐type and p‐type thermoelectric properties are realized, which are much higher than that of the metallic MgNiSb. The proposed bandgap opening strategy can be employed to design and develop new half‐Heusler semiconductors for functional and energy applications.

## Graphical abstract

A d–d orbital interaction‐induced bandgap‐opening strategy is proposed and demonstrated. Semiconducting half‐Heusler compounds can be designed by introducing the transition d‐metals into the metallic half‐Heusler matrix. The obtained semiconducting half‐Heuslers show to possess promising thermoelectric performance.

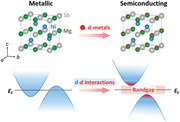

## 1 Introduction

Semiconductors are important and indispensable materials in contemporary human civilized society. The discovery and design of new semiconductors are essential to develop more potential high‐performance functional materials in the field of modern electronics, solar cells, thermoelectrics, and so on. The thermoelectric (TE) technology, which can realize the mutual conversion between heat and electricity, provides a promising solution for self‐powering and precise temperature control in the Internet of Things, 5G communications, and flexible electronics.^[^ ^1^ ^]^ Developing novel materials with good TE performance has attracted considerable attention in the TE community. Among the numerous materials, metals are not potential TE candidates due to their small Seebeck coefficient *S* and high thermal conductivity *κ*, which leads to inferior TE performance with a very low figure of merit, *zT* = *S* ^2^ *σT*/*κ*, where *σ* is the electrical conductivity and *T* is the absolute temperature. On the contrary, many narrow‐gap semiconductors have been found to possess excellent TE performance with *zT* above unity, including V_2_VI_3_, IV‐VI, Zintl phase, and half‐Heusler (HH) compounds.^[^ ^2^ ^]^

HH compounds are a family of materials with the general formula of *XYZ* and space group of *F*‐43*m*, where *X* is the most electropositive metal element, *Y* is the less electropositive metal element, and *Z* is the main group element. In the past decades, several HH semiconductors have been developed as excellent TE materials, for example, ZrNiSn,^[^ ^3^ ^]^ ZrCoSb,^[^ ^4^ ^]^ and NbFeSb,^[^ ^5^ ^]^ and the modules made of these HH materials exhibit both high conversion efficiency and giant power density.^[^ ^6^ ^]^ Among the HH family, many members exhibit metallic behavior due to the absence of a bandgap and thus inferior TE performance, such as MgCuSb and MgNiSb.^[^ ^7^ ^]^ If the bandgap of metallic HHs can be opened, there will be more opportunities to find more potential high‐performance materials and perhaps potential candidates for other functional applications.

**Figure 1:**
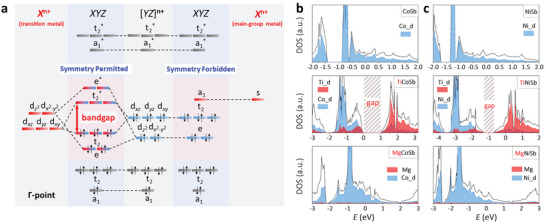
a) Simplified schematic figure of bandgap formation in HHs at the Γ‐point based on molecular orbital theory (electrons assigned assuming VEC = 18), in which when *X* is transition metal, the symmetry‐permitted d–d orbital interaction can open the bandgap of HH *XYZ*, while when *X* is main‐group metal, the symmetry‐forbidden d–s orbital interaction cannot bring about the formation of bandgap; b) the DOS and projected DOS of CoSb, TiCoSb, and MgCoSb; c) the DOS and projected DOS of NiSb, TiNiSb, and MgNiSb.

The origin of the bandgap of HHs is an intriguing topic that has been studied in the past.^[^ ^8^ ^]^ Zintl chemistry has been used to understand the band structure of 18‐electron *XYZ* HHs, in which the *X* element donates all its valence electrons to the covalently bonded zinc‐blende type *YZ* anion framework, where the 18‐electron refers to the total valence electrons count (VEC) of the constituent elements equals to 18, in a stable and semiconductive state.^[^ ^8d^ ^]^ First‐principles calculations have revealed that the conduction band minimum (CBM) and valence band maximum (VBM) in *XYZ* HHs is mainly contributed by the d orbitals of the *X* and *Y* elements,^[^ ^9^ ^]^ which indicates that the d–d orbital interactions between the *X^n^* ^+^ element and the [*YZ*]*^n−^* the framework will be the key to opening the bandgap of HHs. The bandgap formation of HH can be simply understood using the molecular orbital theory, in which the bandgap is formed due to the d–d orbital interactions with mainly d‐orbital contributions from *X* and *Y* elements at the bandgap edges (**Figure** **1**a). The importance of d–d orbital interactions in the bandgap formation can also be recognized from the density of states (DOS) of Ti*YZ* and Mg*YZ* (*YZ* = CoSb or NiSb), shown in Figure 1b,c. For the same *YZ* framework, only the compounds that contain two transition metals, that is, TiCoSb and TiNiSb, have a bandgap. Despite the VEC of TiNiSb not equaling 18, the forbidden d–d energy bandgap can still exist, but its electrical transport deviates from the intrinsic semiconductor behavior owing to the upshift of the Fermi level.^[^ ^10^ ^]^ On the other hand, for the compounds with one transition metal, MgCoSb, and MgNiSb, the energy bandgap does not exist in their electronic band structures near the Fermi level (±1 eV). The projected DOS indicates that there is a negligible contribution of the Mg element in the Mg*YZ* around the Fermi level, in contrast to the large contribution of the Ti element's d orbitals around the bandgap edge of Ti*YZ*. Besides Mg*YZ*‐based HHs, it has also been pointed out in the previous study that the HH compounds with only one transition metal, such as AlNiSb, InPdSb, MgPdTe, etc., are metallic without bandgaps.^[^ ^11^ ^]^

Group theory can provide insight into the metallic behavior in HHs that have only one transition metal. Symmetry is one of the crucial factors that influence orbital interactions.^[^ ^8^, ^12^ ^]^ The symmetry will prohibit the interactions between orbitals that belong to different symmetry representations at a given *k*‐point while permitting the interactions when orbitals belong to the same symmetry representation. As shown in Figure 1a, when forming the molecular orbitals in *XYZ* HHs with one transition metal, the s‐orbitals will not interact with the d‐orbitals at Γ‐point because the s‐orbitals belong to the a_1_ symmetry representation of the *T*_d_ point group, different from the d‐orbitals with t_2_ and e symmetry representation. While for HHs with two transition metals, the symmetry will allow the interactions of d–d orbitals. The molecule orbitals will further form the crystal orbitals (bands) in the solid‐state materials accompanied by the broadening of the orbitals in real space. The crystal orbitals might cross each other and eliminate the bandgap, but at the same time, they may anti‐cross each other if they possess the same symmetry. The anti‐crossings or avoided crossings in reciprocal space at different *k* points have been developed to elucidate the bandgap formation and band degeneracy in transition‐metal HHs in prior works.^[^ ^8a,e^ ^]^

Besides symmetry, the energy of s‐orbitals relative to d‐orbitals is also an important factor that may influence the existence or size of a bandgap. ScPtBi and HfIrBi are examples that do not have the bandgap despite the d–d orbital interactions that are present in the *XYZ* HH matrix because of their low‐lying s‐orbital energy of *Z* elements.^[^ ^11^ ^]^ On the other hand, a bandgap could also exist in the compounds when the *X* element s‐orbital energy *E*(*X*‐s) is high enough relative to the d‐orbitals of *Y* element, as shown in Figure S1a, Supporting Information, and the bandgap size will increase with increasing *E*(*X*‐s). But when *E*(*X*‐s) is lowered, the formed s‐ and d‐bands will cross at Γ point (Figure S1b, Supporting Information), where they are non‐interacting due to their different symmetry representations. Eventually, the s‐band will be lower than the d‐band (Figure S1c, Supporting Information), but the band crossing at Γ will still be present, leading to no bandgap. In most HHs with the main group metal occupying the *X*‐site, such as MgNiSb and AlNiSb, *E*(*X*‐s) is relatively low relative to *E*(*Y*‐d), which will eliminate the bandgap because the s–d orbital interactions are symmetry forbidden at the Γ‐point.

Typically, these metallic HHs are not good TE candidates due to their inferior Seebeck coefficient, so engineering the band structure to open the bandgap is an effective way to improve their TE performance. Since the 1990s, band engineering strategies, including quantum well,^[^ ^13^ ^]^ resonant level,^[^ ^14^ ^]^ band convergence,^[^ ^15^ ^]^ band sharpening,^[^ ^16^ ^]^ and band anisotropy,^[^ ^17^ ^]^ have been proposed to boost the performance of TE materials.^[^ ^2^ ^]^ Based on the bandgap formation mechanism above, introducing the d–d orbital interactions in the metallic HH matrix will be a feasible way to open the bandgap. However, there is little research on introducing d–d orbital interactions via alloying as a means to open the bandgap in HHs, which motivates us to carry out this study.

In this work, we propose a bandgap opening strategy that involves introducing d–d orbital interactions by alloying on the HH *X*‐site with a d‐block transition metal. By taking metallic MgNiSb as an example, we found that the addition of a transition metal in MgNiSb can introduce d–d orbital interactions and open the bandgap. The theoretical and experimental studies herein further reveal a strong bandgap dependence on d–d orbital interactions. By optimizing the Ti concentration determining the bandgap energy and the carrier type/density, the TE performance of both n‐ and p‐type Mg_1‐_ *_x_* Ti*_x_* NiSb are largely improved. The proposed bandgap‐opening strategy enables the design of metallic HHs for potential TE materials and will probably inspire the development of other semiconductors with transition metals.

## 2 Results and Discussion

### 2.1 Introducing d–d Orbital Interactions

The HH MgNiSb is taken as the study object based on the previous study,^[^ ^7a^ ^]^ in which MgNiSb is reported to be a metal with very small positive *S*, the negative temperature dependence of *σ*, and inferior TE performance *zT* ≈ 0.00057. Our experiments of MgNiSb also confirm its metallic nature and very poor TE performance with *zT* of about 10^−4^ (Figure S2, Supporting Information). The band structure calculation also verifies the non‐existence of bandgap in MgNiSb (Figure S3, Supporting Information). Based on the bandgap formation principles of HHs mentioned above, the lack of a bandgap of MgNiSb can be ascribed to the absence of d–d orbital interactions between Mg and Ni. The Mg s‐orbital dominated band will cross the d‐orbital bands of Ni due to the different symmetry representations of the s and d‐orbitals at the Γ point, which therefore results in the lack of bandgap in MgNiSb. Introducing the d–d orbital interactions in MgNiSb may be an effective strategy to open its bandgap and enhance its TE performance.

**Figure 2:**
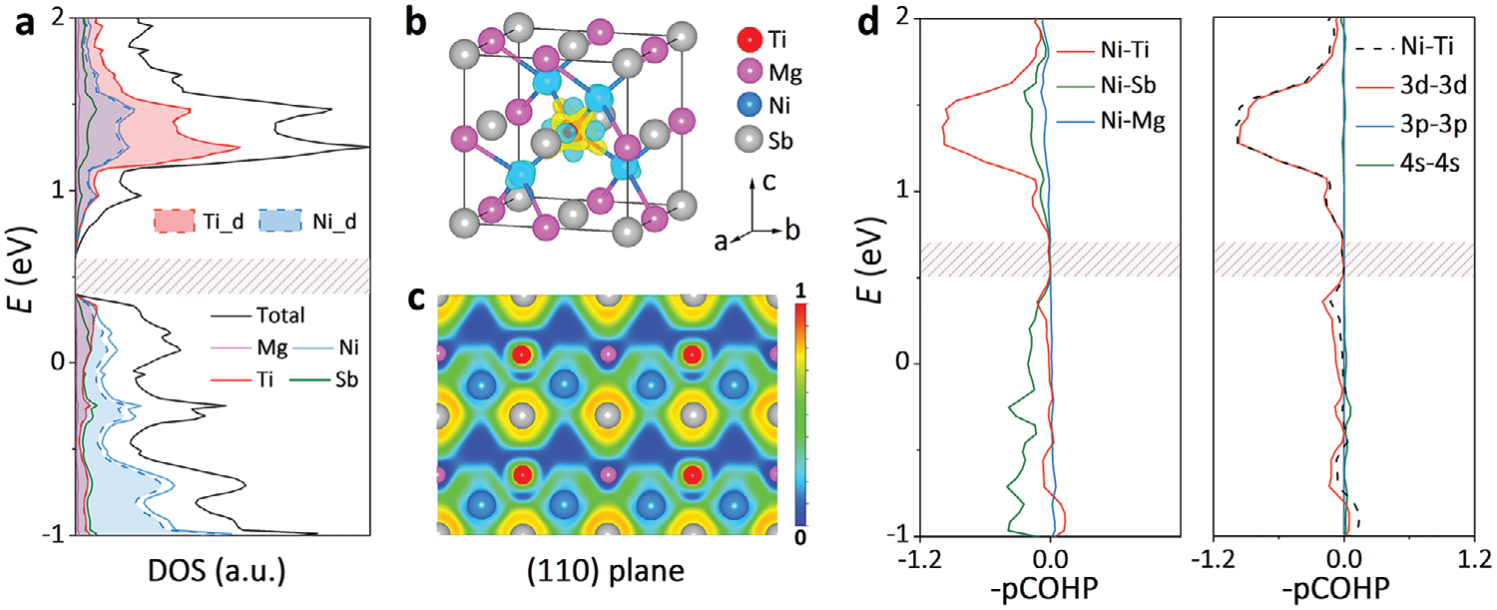
a) The total DOS and projected DOS, b) DCD, in which the isosurface level is taken as 1.9 × 10^−2^ *e* Å^−3^; c) ELF in the (110) plane, where the blue color with ELF = 0 indicates the electrons are totally delocalized surrounding Mg atoms and the green color with ELF = 0.5 indicates the electrons are paired surrounding Ti, Ni, and Sb; d) the pCOHP of Mg_0.75_Ti_0.25_NiSb.

Adding a d‐block transition metal at the Mg site is a direct way to incorporate d‐orbitals and hence introduce d–d orbital interactions into the matrix. Here, we first take Ti‐substituted MgNiSb as an example to validate this idea. When one‐quarter of Mg is substituted by Ti in Mg_1‐_ *_x_* Ti*_x_* NiSb, the calculated DOS of Mg_0.75_Ti_0.25_NiSb in **Figure** **2**a indicates the existence of a bandgap. The projected DOS further reveals that CBM and VBM of Mg_0.75_Ti_0.25_NiSb are mainly composed of the d orbitals of Ti and Ni with the negligible contribution of Mg. This means that the added transition‐metal Ti in Mg_0.75_Ti_0.25_NiSb successfully induces the d–d orbital interactions between Ni and Ti into the matrix and opens the bandgap.

To further probe the d–d orbital interactions of Ni and Ti, it is intuitive to investigate the electronic structure in real space. The deformation charge density (DCD) has been calculated first and is shown in Figure 2b. It can be found that the accumulation and depletion of charge density only happen around Ti and Ni, indicating the interactions of Ti and Ni. The electron localization function (ELF) in the (110) plane (Figure 2c) further shows that the electrons are delocalized around the Mg elements (blue color with ELF = 0), but are paired surrounding Ti, Ni, and Sb (green color with ELF = 0.5). This result is consistent with the concept of Zintl chemistry mentioned above, in which both Ti and Mg donate electrons to the NiSb framework, but only Ti will interact with the NiSb framework through d–d orbital interactions. The interaction features in Mg_1‐_ *_x_* Ti*_x_* NiSb can also be seen from the projected crystal orbital Hamilton population (pCOHP) in Figure 2d and Figure S4, Supporting Information.^[^ ^18^ ^]^ Both the interactions of the nearest Ni‐Sb and Ni‐Ti are found at the bandgap edges with negligible Ni–Mg interactions. Importantly, it can be seen that the interactions of Ni–Ti are mainly composed of the d–d orbital interactions. This means that the addition of transition‐metal Ti in MgNiSb introduces mainly d–d orbital interactions of Ni and Ti in the matrix, and therefore opens the bandgap.

### 2.2 Opening the Bandgap of Metallic HHs

**Figure 3:**
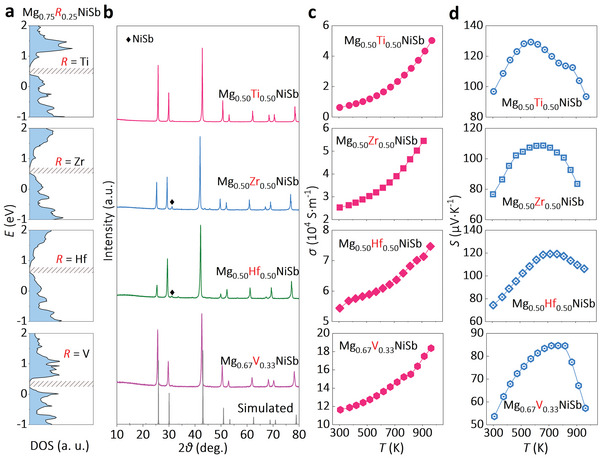
a) The DOS and b) XRD patterns of Mg_1‐_ *_x_R_x_* NiSb (*R* = Ti, Zr, Hf, and V); temperature dependences of c) *σ* and d) *S* of Mg_1‐_ *_x_R_x_* NiSb (*R* = Ti, Zr, Hf, and V).

Based on the above first‐principles calculations, the addition of transition‐metal Ti in MgNiSb induces the d–d orbital interactions in the matrix and opens the bandgap. In addition to Ti, other transition metals, such as Zr, Hf, and V, can also introduce d–d orbital interactions in Mg_0.75_ *R*_0.25_NiSb (*R* = Ti, Zr, Hf, V, etc.) and open the bandgap, as displayed in calculated DOS (**Figure** **3**a and Figure S5, Supporting Information).

This band‐gap opening strategy is further verified experimentally. The VEC of the materials Mg_1‐_ *_x_R_x_* NiSb (*R* = Ti, Zr, Hf, and V) has been fixed to 18 so that the effect of aliovalent doping by these transition metals is excluded. Mg_0.5_Ti_0.5_NiSb, Mg_0.5_Zr_0.5_NiSb, Mg_0.5_Hf_0.5_NiSb, and Mg_0.67_V_0.33_NiSb have all been synthesized by solid‐state reactions (see Experimental Section, Supporting Information). These compounds have been referred to as “double HH” and “triple HH” compounds in recent calculation works.^[^ ^19^ ^]^ The X‐ray diffraction (XRD) patterns of the synthesized samples confirm their HH crystal structures, as displayed in Figure 3b. Some NiSb impurities could be detected as well, which might arise from the loss of Mg during synthesis. The Rietveld analysis of the XRD data in Figure S6, Supporting Information indicates that these Mg‐based HH do not have a large number of Mg vacancies, which means the loss of Mg during synthesis just results in the formation of NiSb impurities in the matrix. Figure 3c displays the measured *σ* of the synthesized materials in the range of 300–900 K, and all of them exhibit a typical semiconducting behavior. The bandgap *E*_g_ of these compounds can be estimated according to either *σ* ^−1^ = *σ*_0_·exp(*E*_g_/2*kT*) or Goldsmid‐Sharp formula *E*_g_ = 2*eS*_max_ *T*_max_,^[^ ^20^ ^]^ where *σ*_0_ is a constant, *k* is Boltzmann constant, *e* is the elemental charge, *S*_max_ is the maximum value of *S*, and *T*_max_ is the corresponding temperature of *S*_max_. For Mg_0.5_Ti_0.5_NiSb, the bandgap is estimated to be about 0.22 eV and 0.15 eV from the measured *σ* and *S* in Figure 3c,d, respectively. Both of them are smaller than the calculated ones (0.3 eV in this work, 0.47 eV according to Anand et al.^[^ ^19a^ ^]^). This discrepancy might arise from poorer weighted mobility for the underestimated Goldsmid gap on the one hand.^[^ ^21^ ^]^ Additionally, the small experimental bandgap may also be due to the presence of an impurity Ni interstitial band like that found in ZrNiSn.^[^ ^22^ ^]^ Scanning transmission electron microscopy (STEM) has therefore been adopted to investigate the atomic scale microstructure of Mg_0.5_Ti_0.5_NiSb semiconductors.

**Figure 4:**
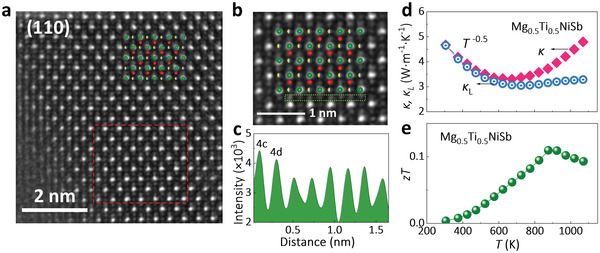
a) The HAADF image in (110) plane of Mg_0.5_Ti_0.5_NiSb; b) the magnified HAADF image of (a) in red dashed box, and the crystal structure of half‐Heusler is inserted; c) the intensity profile taken along the 4c/4d plane as indicated by the green dashed box in (b); temperature dependences of d) *κ* and *κ*_L_, and e) *zT* of Mg_0.5_Ti_0.5_NiSb.

As shown in **Figure** **4**a,b, the high‐angle annular dark‐field (HAADF) images of Mg_0.5_Ti_0.5_NiSb in the (110) plane indicate that some atoms occupy the interstitial 4d (Wyckoff position) sites. An alternating intensity profile within the 4c/4d plane (Figure 4c) also suggests that the 4d sites have been partially occupied, similar to the phenomenon observed in ZrNiSn with Ni 4d interstitials and ZrCoSb with Co 4d interstitials.^[^ ^23^ ^]^ These interstitial atoms might bring the in‐gap states and lead to a smaller observable bandgap than the first‐principles calculations.^[^ ^8c^ ^]^ The energy dispersive spectroscopy (EDS) mappings of Mg, Ti, and Sb in Figure S7, Supporting Information show that these elements all occupy the sites in the HH structure with Mg and Ti at the same 4a site and Sb at the 4b site. The mixture of Mg/Ti, as well as the 4d interstitials, can bring about strong point defects scattering of phonons, which will significantly influence the thermal transport of the material. As shown in Figure 4d, the *κ* and lattice thermal conductivity *κ*_L_, obtained by subtracting electronic thermal conductivity *κ*_e_ = *LσT* from *κ*, where *L* is Lorenz number, calculated based on single parabolic band model,^[^ ^24^ ^]^ of Mg_0.5_Ti_0.5_NiSb is 5 W·m^−1^·K^−1^ at room temperature, much lower than the conventional HH compounds, such as ZrNiSn ≈8–10 W·m^−1^·K^−1^ and NbFeSb ≈10–20 W·m^−1^·K^−1^.^[^ ^25^ ^]^ Furthermore, the temperature dependence of *κ*_L_ shows a tendency about *T* ^−0.5^, implying that point defects may dominate the phonon scattering. The *zT* value of Mg_0.5_Ti_0.5_NiSb is estimated to be 0.11 at 875 K (Figure 4e), which is not high, but still exhibits 200 times increase compared with the metallic MgNiSb matrix.

### 2.3 Bandgap Variation and p‐n Transition

The effectiveness of introducing d–d interactions as a bandgap‐opening strategy in metallic MgNiSb has been verified theoretically and experimentally. This strategy can also be explored in other metallic HHs. For example, it is found that the bandgap can be opened in both MgNiSn‐based, MgCoSb‐based, and MgPdSb‐based HHs when the transition‐metal d–d interactions exist (see Figure S8, Supporting Information). These new HH semiconductors have not yet been discovered or reported before, and they may be potential TE candidates as well. These results motivate us to think about another question, that is, the relation between the width of the bandgap and the strength of d–d interactions, which is positively proportional to the content of the introduced transition‐metal.

**Figure 5:**
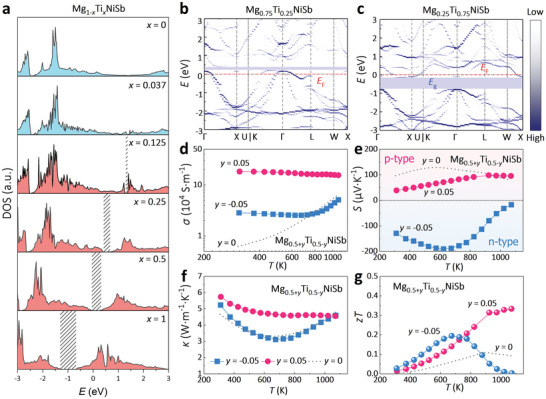
a) The DOS of Mg_1‐_ *_x_* Ti*_x_* NiSb with different Ti contents, where the Fermi level *E*_F_ is located at 0 eV; the unfolding band structures of b) Mg_0.25_Ti_0.75_NiSb and c) Mg_0.75_Ti_0.25_NiSb; temperature dependences of d) *σ*, e) *S*, f) *κ*, and g) *zT* of the Mg_0.5+_ *_y_* Ti_0.5‐_ *_y_* NiSb (*y* = −0.05 and 0.05).

As shown in **Figure** **5**a, it is found that in Mg_1‐_ *_x_* Ti*_x_* NiSb, the higher the Ti content, the stronger the d–d interactions and the larger the bandgap. An obvious bandgap of 0.06 eV can be obtained when the Ti content is about 0.125, but it almost vanishes when the Ti content is about 0.037 (the enlarged figure is shown in Figure S9, Supporting Information). Therefore, there should be a critical value related to the d–d orbital interactions (the content of Ti) so that the bandgap of Mg_1‐_ *_x_* Ti*_x_* NiSb is opened, which is in the range of 0.037–0.125. It is worth noting that the critical value for opening the bandgap will vary if different transition‐metal elements are introduced into MgNiSb. As shown in Figure S5, Supporting Information, different transition metals result in different bandgaps when the contents of transition‐metal *x* are fixed to 0.25 in Mg_1‐_ *_x_R_x_* NiSb.

The dependence of bandgap on d–d orbital interactions in HHs also provides a means to tune the bandgap, which is critical for developing TE semiconductors with different optimal operating temperatures. This bandgap engineering in HHs can easily be realized by adjusting the content of the transition metal in the matrix. In addition to the widened bandgap, it is also found that the Fermi level *E*_F_ will move from the valence band to the conduction band with increasing Ti content (Figure 5b,c), indicating a change of carrier density and eventually conduction behavior. These evolutions are caused by the aliovalent doping from the transition metal. MgNiSb has a VEC of 17, while it is 19 for TiNiSb. Therefore, when the Ti content *x* is smaller than 0.5, the compound has excess holes and acts as a p‐type semiconductor while it will behave like an n‐type semiconductor if *x* > 0.5, owing to the excess electrons. This has been verified in experiments (Figure 5d). When the content of Ti deviates from 0.5, the *σ* of both Ti‐rich (*y* = −0.05) and Ti‐poor (*y* = 0.05) samples increases as a result of the shift of *E*_F_ from the forbidden gap toward the conduction and valence bands, respectively. This also yields the negative *S* in the Ti‐rich sample and the positive *S* in the Ti‐poor sample (Figure 5e). The realization of both n‐type and p‐type conduction behaviors in the same matrix by only adjusting the Ti content is desired for designing n‐type and p‐type legs from the view of TE devices as they will show very close thermal expansion. Combining *σ*, *S*, and *κ* (Figure 5f), the *zT* values reach 0.33 at 1070 K and 0.19 at 674 K for n‐type and p‐type, respectively (Figure 5g).

### 2.4 Optimizing TE Performance

**Figure 6:**
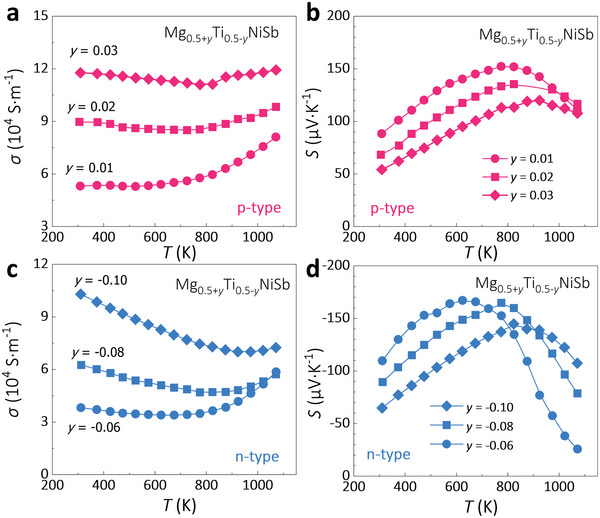
Temperature dependences of a) *σ* and b) *S* of p‐type Mg_0.5+_ *_y_* Ti_0.5‐_ *_y_* NiSb (*y* = 0.01, 0.02 and 0.03), c) *σ* and d) *S* of n‐type Mg_0.5+_ *_y_* Ti_0.5‐_ *_y_* NiSb (*y* = −0.06, −0.08 and −0.10).

Adjusting the content of transition metal on the *X*‐site of HHs not only tunes the bandgap energy but also modifies the carrier concentration and conduction type. Therefore, the alloying content of transition metal, despite tuning the bandgap, might also be detrimental (or beneficial) to the TE performance in terms of the optimization of carrier concentration. To reach the optimal TE performance in Mg_1‐_ *_x_* Ti*_x_* NiSb (both n‐type and p‐type), one should consider the changes in both the bandgap and carrier type/concentration when adjusting the Ti content. It is recognized that the bandgap shrinks with decreasing Ti content (Figure 5a) and thus, the variation of Ti contents for tuning the carrier density should be smaller for Ti‐poor (p‐type) samples relative to Ti‐rich (n‐type) samples. A series of Mg_0.5+_ *_y_* Ti_0.5‐_ *_y_* NiSb (*y* = 0.01, 0.02, 0.03 for p‐type Ti‐poor samples and *y* = −0.06, −0.08, −0.10 for n‐type Ti‐rich samples) have been synthesized. For p‐type samples, the *σ* increases while *S* reduces with decreasing Ti content (**Figure** **6**a,b). This is a result of the enhanced hole concentration with the VEC approaching 17. Similarly, for the n‐type samples (Figure 6,d), the *σ* increases while *S* decreases when the Ti content deviates from 0.5 due to the enhanced electron concentration with VEC approaching 19. Meanwhile, it can be seen in Figure 6b,d that the bipolar effects of both n‐type and p‐type are suppressed and shifted to high temperatures caused by the increased holes and electrons, respectively.

**Figure 7:**
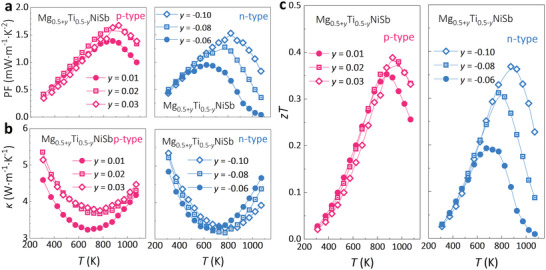
Temperature dependencies of a) PF and b) *κ*, and c) *zT* of p‐type Mg_0.5+_ *_y_* Ti_0.5‐_ *_y_* NiSb (*y* = 0.01, 0.02 and 0.03) and n‐type Mg_0.5+_ *_y_* Ti_0.5‐_ *_y_* NiSb (*y* = −0.06, −0.08 and −0.10).

The power factor PF = *S* ^2^ *σ* of both n‐type and p‐type can reach 1.5 mW·m^−1^·K^−2^, as shown in **Figure** **7**a. The *κ* of both n‐type and p‐type is within the range of 3 to 5.5 W·m^−1^·K^−1^ from 300 K to 1073 K (Figure 7b), indicating its intrinsic low thermal conductivity, which is caused by the strong point defect scattering of phonons. As a result, the peak *zT* of both n‐type and p‐type samples can reach about 0.4 (Figure 7c). Even though their TE performance is not as good as n‐type ZrNiSn and p‐type NbFeSb,^[^ ^25^, ^26^ ^]^ these newly developed Mg‐based HH compounds have advantages for potential TE applications, including the adjustable bandgap benefiting for a wide working temperature range, very close matrix composition for n‐type and p‐type legs, and low cost. Here, we demonstrated the TE performance optimization of Mg_0.5_Ti_0.5_NiSb by changing the Ti contents, which tunes the bandgap and Fermi level simultaneously. In future studies, the TE performance might be further improved by wisely doping with fixed bandgap and using currently developed strategies, such as phonon engineering, including hierarchical phonon scattering and selective scattering of phonons,^[^ ^4^, ^5^ ^]^ or band engineering, including band sharpening and band convergence.^[^ ^16^, ^17^ ^]^

## 3 Conclusion

In summary, a bandgap opening strategy by the introduction of d–d orbital interactions has been proposed and verified in MgNiSb‐based HH materials. The addition of a transition‐metal in metallic MgNiSb can introduce d–d orbital interactions in the material and thus open the bandgap. Mg_1‐_ *_x_R_x_* NiSb (*X* = Ti, Zr, Hf, V) compounds have been successfully synthesized, and all of them can exhibit semiconducting behaviors, indicating the effectiveness of introducing d–d orbital interactions to open the bandgap. Among these newly developed semiconductors, Mg_0.5_Ti_0.5_NiSb is estimated to have a bandgap of about 0.15 eV based on the Seebeck coefficient *S*. The HAADF images and EDS mappings show that Mg_0.5_Ti_0.5_NiSb has an Mg/Ti mixture and interstitials, which brings about strong point defect phonon scattering and results in a relatively low thermal conductivity (about 5 W·m^−1^·K^−1^ at 300 K). As a result, the *zT* of Mg_0.5_Ti_0.5_NiSb is 200 times higher than that of metallic MgNiSb. This bandgap opening strategy can also be applied to other metallic HHs that only have one transition metal, paving the way to design and develop new HH semiconductors.

Furthermore, it is found the bandgap has a strong dependence on the d–d orbital interactions in HHs. A critical strength of d–d orbital interactions is needed to open the bandgap of the metallic HHs, and the stronger the d–d orbital interactions, the larger the bandgap. In addition to the bandgap variation, it is found that the carrier density and even conduction type will also change when increasing the content of transition metal and d–d interactions. The variation in the bandgap and conduction type facilitates the development of TE materials applied at different temperatures and with both n‐type and p‐type transport behaviors. Balancing the variation in the bandgap and carrier density, the TE performance of Mg_1‐_ *_x_* Ti*_x_* NiSb is optimized by tuning the Ti content, giving a decent peak *zT* of about 0.4 in both n‐type and p‐type samples. This work demonstrates the tuning of metallic half‐Heuslers to semiconductors through the d–d interactions‐induced bandgap‐opening strategy, which will help to discover more new semiconductors for potential functional and energy applications.

## Conflict of Interest

The authors declare no conflict of interest.

## Supporting information

Supporting Information

## Data Availability

The data that support the findings of this study are available from the corresponding author upon reasonable request.
