# Supplementary material for: Opening the Bandgap of Metallic Half‐Heuslers via the Introduction of d–d Orbital Interactions
Source: Adv Sci (Weinh). 2023 Jun 4;10(23):2302086. doi: 10.1002/advs.202302086 (PMC10427359; doi:10.1002/advs.202302086)
Supplement: Supplementary file 1 — Supporting Information [file ADVS-10-2302086-s001.pdf]

ADVANCED SCIENCE
Open Access

Supporting Information

for *Adv. Sci.*, DOI 10.1002/advs.202302086

Opening the Bandgap of Metallic Half-Heuslers via the Introduction of d–d Orbital Interactions

Airan Li, Madison K. Brod, Yuechu Wang, Kejun Hu, Pengfei Nan, Shen Han, Ziheng Gao, Xinbing Zhao, Binghui Ge, Chenguang Fu, Shashwat Anand, G. Jeffrey Snyder and Tiejun Zhu**

Supporting Information

Opening the Bandgap of Metallic Half-Heuslers via the Introduction of d-d Orbital Interactions

Airan Li,[a] Madison K. Brod,[b] Yuechu Wang,[a] Kejun Hu,[c] Pengfei Nan,[c] Shen Han,[a] Ziheng Gao,[a] Xinbing Zhao,[a] Binghui Ge,[c] Chenguang Fu,*[a] Shashwat Anand,[d] G. Jeffrey Snyder,[b] Tiejun Zhu*[a]

[a] A. R. Li, Y. C. Wang, S. Han, Z. H. Gao, Dr. X. B. Zhao, Dr. C. G. Fu, Dr. T. J. Zhu
State Key Laboratory of Silicon Materials, School of Materials Science and Engineering
Zhejiang University
Hangzhou 310058, China
E-mail: chenguang_fu@zju.edu.cn, zhutj@zju.edu.cn

[b] M. K. Brod, G. J. Snyder
Department of Materials Science and Engineering
Northwestern University
Evanston, Illinois 60208, United States

[c] K. J. Hu, Dr. P. F. Nan, Dr. B. H. Ge
Information Materials and Intelligent Sensing Laboratory of Anhui Province, Key Laboratory of Structure and Functional Regulation of Hybrid Materials of Ministry of Education, Institutes of Physical Science and Information Technology
Anhui University
Hefei 230601, China

[d] Dr. S. Anand
Materials Sciences Division
Lawrence Berkeley National Laboratory
Berkeley, California 94720, United States

Table of Contents

1. Experimental Procedures....................3

Materials synthesis....................3

Characterization & Measurement....................3

First-principles calculations....................3

2. Supplementary Figures....................4

Figure S1. Simplified sketch of the band structure in half-Heusler compounds....................4

Figure S2. Themoelectric transport property of MgNiSb....................5

Figure S3. Band structure of MgNiSb....................6

Figure S4. The pCOHP of $Mg_{0.75}Ti_{0.25}NiSb$....................7

Figure S5. Density of states of $Mg_{0.75}M_{0.25}NiSb$....................8

Figure S6. Rietveld analysis of $Mg_{0.5}Ti_{0.5}NiSb$, $Mg_{0.5}Zr_{0.5}NiSb$, $Mg_{0.5}Hf_{0.5}NiSb$, $Mg_{0.67}V_{0.33}NiSb$....................9

Figure S7. EDS mappings of elements in $Mg_{0.5}Ti_{0.5}NiSb$....................10

Figure S8. Density of states of $Mg_{0.75}R_{0.25}NiSn$, $Mg_{0.75}R_{0.25}CoSb$, $Mg_{0.75}R_{0.25}PdSb$....................11

Figure S9. Density of states of $Mg_{0.875}Ti_{0.125}NiSb$ and $Mg_{0.963}Ti_{0.037}NiSb$. $Mg_{0.875}Ti_{0.125}NiSb$....................12

3. References....................13

4. Authors Contributions..13

1. Experimental Procedures

Materials synthesis. $Mg_{0.5+y}Ti_{0.5-y}NiSb$ (y = -0.10, -0.08, -0.06, -0.05, 0, 0.01, 0.02, 0.03, 0.05), $Mg_{0.5}Zr_{0.5}NiSb$, $Mg_{0.5}Hf_{0.5}NiSb$, $Mg_{0.67}V_{0.33}NiSb$ and $Mg_{0.75}Cr_{0.25}NiSb$ were synthesized with starting elements Mg (powders, 99.8%), Ti (powders, 99.9%), Zr (powders, 99.9%), Hf (powders, 99.9%), V (powders, 99.9%), Ni (powders, 99.9%) and Sb (powders, 99.9%). The raw material powders were weighed nominally and mixed in the mortar in the glove box, and then sealed in Ta tubes under the argon atmosphere. The Ta tubes were then sealed in the quartz tube, put into the furnace, heated up to 1273 K, and kept for 24 h for solid-state reaction. The obtained materials were ball-milled (SPEX-8000D; PYNN) for 0.5 h and then loaded into graphite dies (diameter: 12.7 mm). The spark plasma sintering (SPS) (LABOX-650F, Sinter Land Inc.) under 1073 K and 50 MPa for 2 min was carried out to get the bulk form of the material for further characterization and measurement.

Characterization & Measurement. The phases and purities of the obtained materials were checked by the XRD technique (PANalytical, Aries DY866, Cu Kα). The microstructure of $Mg_{0.5}Ti_{0.5}NiSb$ was investigated by the STEM (Cs corrected JEOL ARM 200F microscope, 200 kV). Specimens for the observation were prepared according to previous methods.[1] The EDS mappings of the constituent elements were also carried out (FEI Titan Themis Z microscope equipped with probe and image correctors, 300 kV) to study the atom positions. The Seebeck coefficient S and the electrical conductivity σ of all samples were measured by the commercialized equipment (Linseis LSR-3 system). The measurement uncertainties of S and σ is about 5% and 3% respectively. Thermal conductivity κ was calculated using the equation: $\kappa = D\rho C_p$, where ρ is the sample density measured by the Archimedes method, and C_p is the heat capacity, which was estimated from the Dulong-Petit law, and D is the thermal diffusivity, which was measured by a laser flash method (Netzsch LFA457). The accuracy of κ is about ±10%.

First-principles calculations. The first-principles calculations based on density functional theory were realized by the software, the Vienna ab initio Simulation Package (VASP) with the projector augmented wave method.[2] Both generalized gradient approximation-Perdew-Burke-Ernzerhof type (GGA-PBE),[3] and the Heyd-Scuseria-Ernzerhof (HSE) [4] screened hybrid functional was used as the exchange-correlation functional. Plane-wave cutoff energy was set as 500 eV, and the atomic positions of materials were relaxed until the calculated Hellmann-Feynman force was less than 0.001 eV/Å. The energy convergence criterion for geometry relaxation and self-consist single-point calculation was set to be 10^{-7} and 10^{-8} eV, respectively. The k-point sampling for geometry relaxation and self-consistent static calculations was set to be $k = 30/L$ and $60/L$ (L is the corresponding lattice parameter) with a Gamma-centered scheme, respectively. VASPKIT [5] and BandUP [6] have been used to post-processing the calculated data. **Figure S1** in supplemental information is made by using the tight-binding model based on previous studies.[7]

2. Supplementary Figures

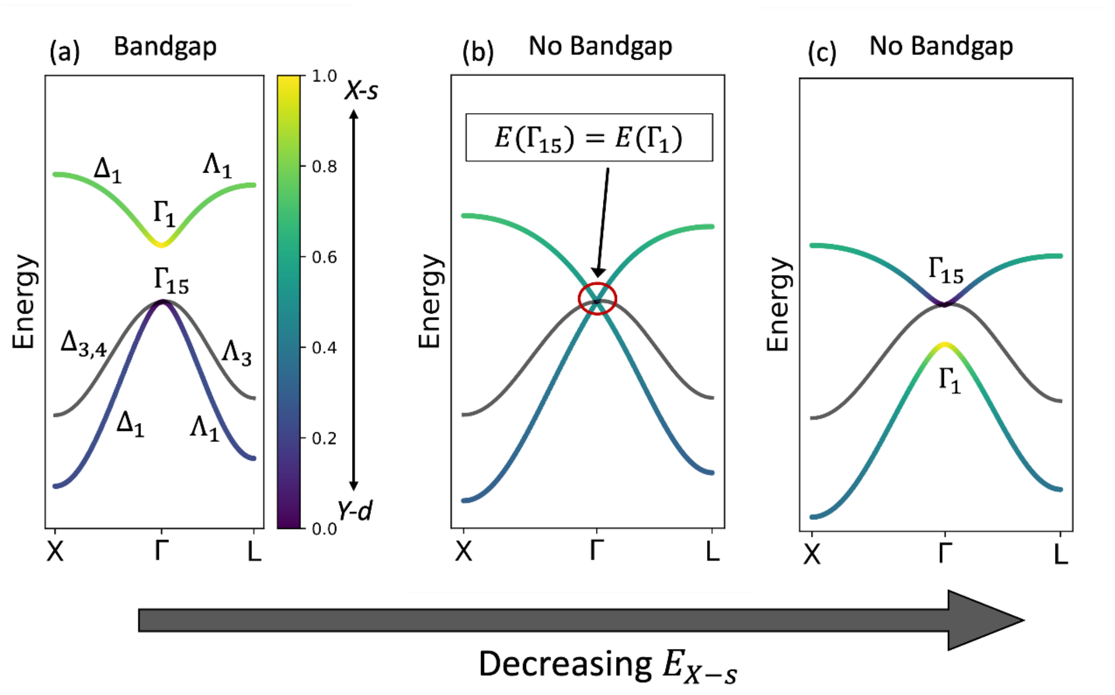

Figure S1. Simplified sketch of the band structure in HHs compounds with *X* s-orbital conduction band and *Y* d-orbital valence bands. The singly degenerate bands are colored to reflect the s-orbital contribution, while the doubly degenerate VB is colored dark gray. (a) When the energy of the *X*-s orbital (*E*(*X*-s)) is sufficiently high relative to the conduction band edge, there is a bandgap, and the bandgap energy will increase with increasing *E*(*X*-s). (b) The conduction band crosses the valence band, eliminating the bandgap once the energy of the Γ_{15} (d_{xy}, d_{yz}, d_{zx}) band equals the energy of the Γ_1 (s) band. (c) If E (*X*-s) is lowered more, then band inversion will occur, causing the Γ_1 band to be below the Γ_{15} band.

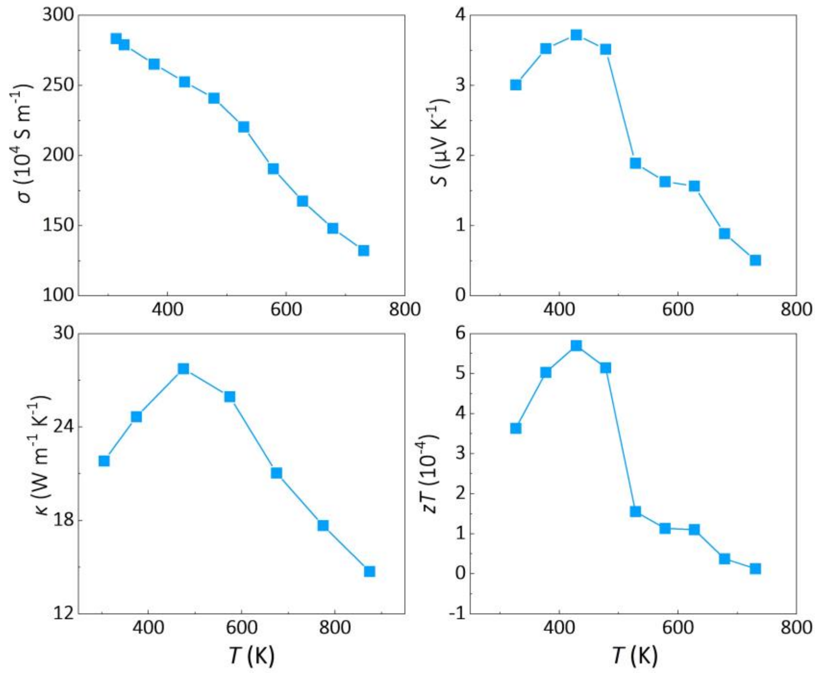

Figure S2. The thermoelectric performance of MgNiSb without substitution. It has a very low Seebeck coefficient and figure of merit *zT*.

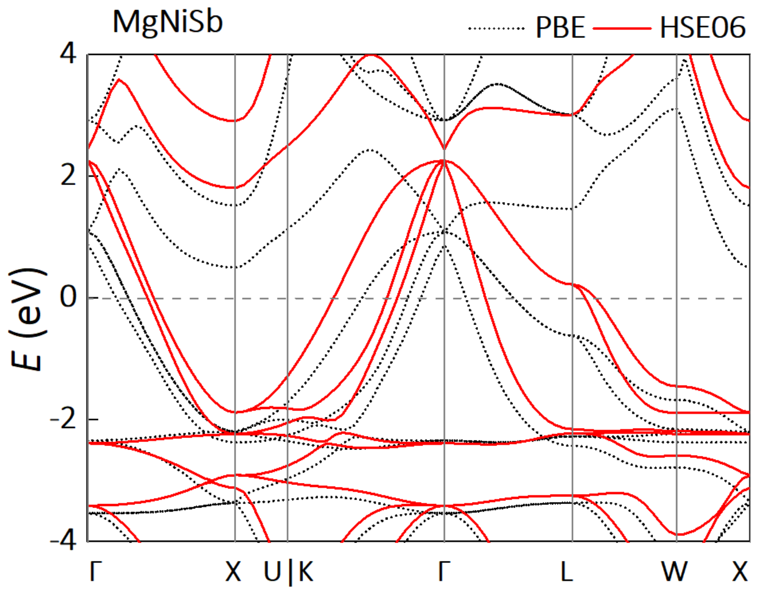

Figure S3. The band structure of MgNiSb with PBE functional and HSE06 hybrid functional.

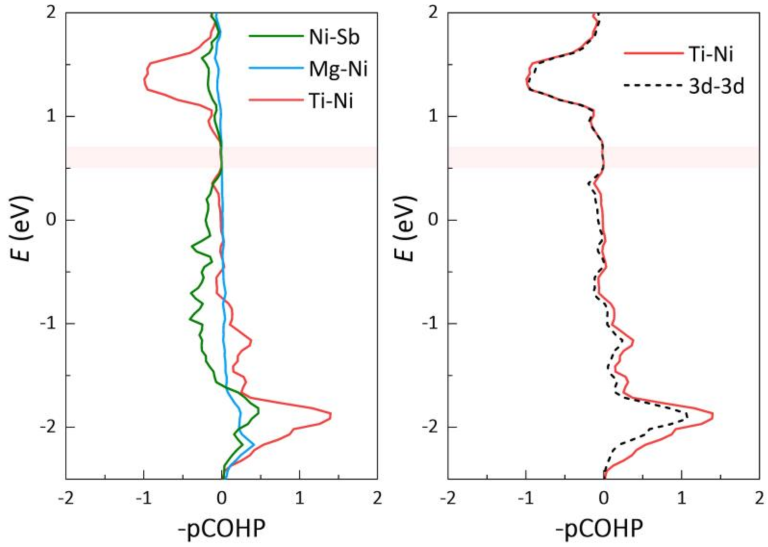

Figure S4. The pCOHP of $Mg_{0.75}Ti_{0.25}NiSb$ in the energy range from -2.5 eV to 2 eV. Its bonding and antibonding states can be found at around -2 eV and 1.4 eV, respectively

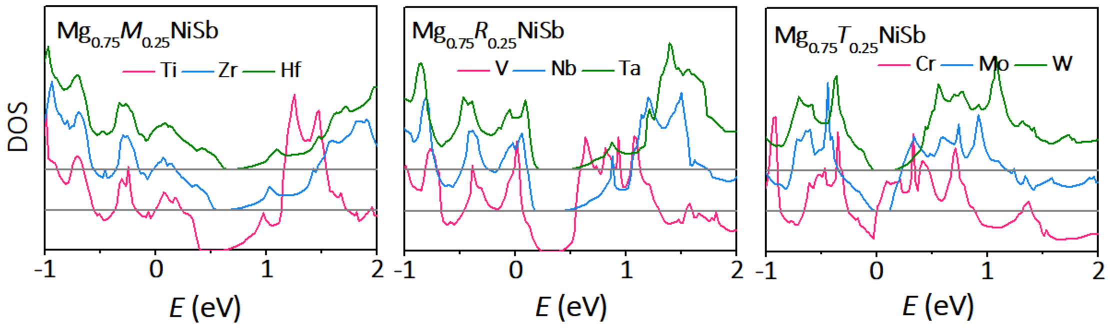

Figure S5. The density of states of $Mg_{0.75}M_{0.25}NiSb$ (*M* = Ti, Zr, Hf), $Mg_{0.75}R_{0.25}NiSb$ (*R* = V, Nb, Ta), $Mg_{0.75}T_{0.25}NiSb$ (*T* = Cr, Mo, W). Both of them have the energy bandgap except $Mg_{0.75}Cr_{0.25}NiSb$, which might originate from the first-principles calculations with PBE functional.

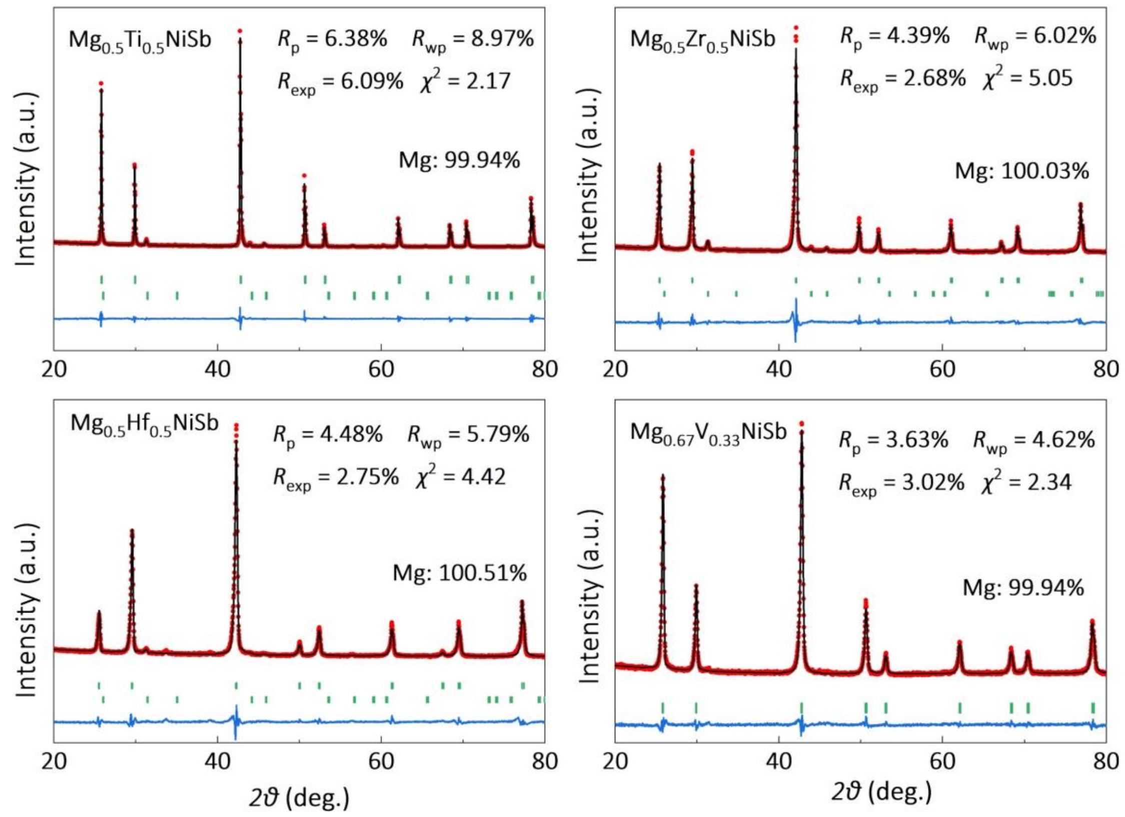

Figure S6. The Rietveld analysis of XRD data of $Mg_{0.5}Ti_{0.5}NiSb$, $Mg_{0.5}Zr_{0.5}NiSb$, $Mg_{0.5}Hf_{0.5}NiSb$, $Mg_{0.67}V_{0.33}NiSb$. Both of them have Mg occupancy of around 100%, which implies that there might be no serious Mg vacancies in the matrix.

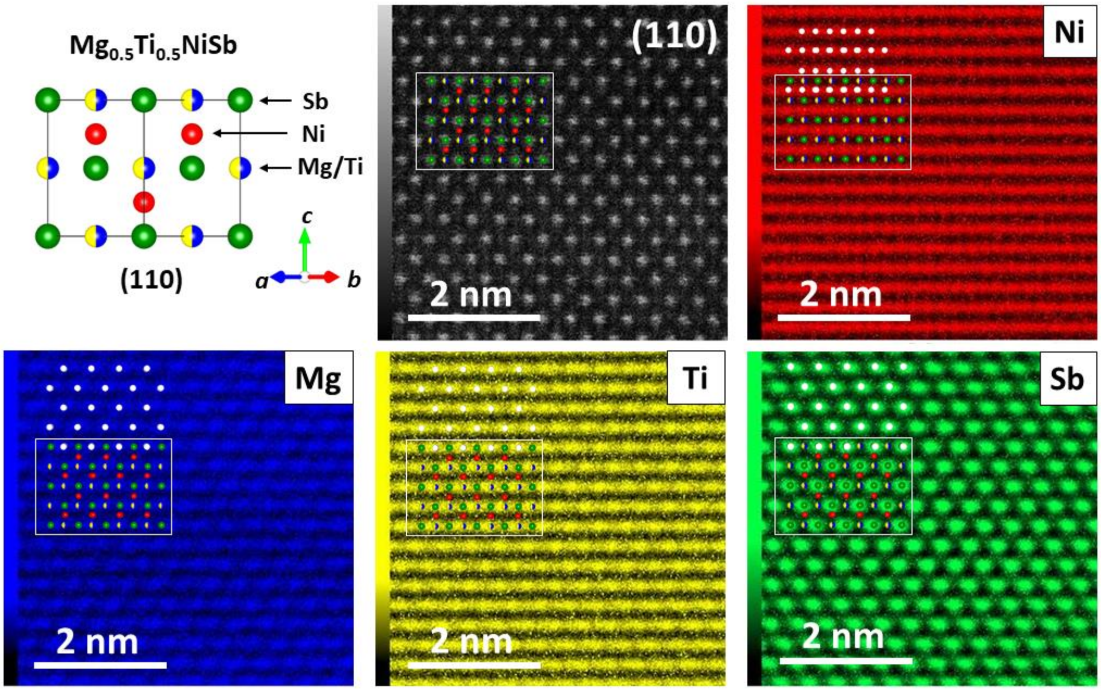

Figure S7. The crystal structure, HAADF image and corresponding EDS mappings of elements in the (110) plane of $Mg_{0.5}Ti_{0.5}NiSb$.

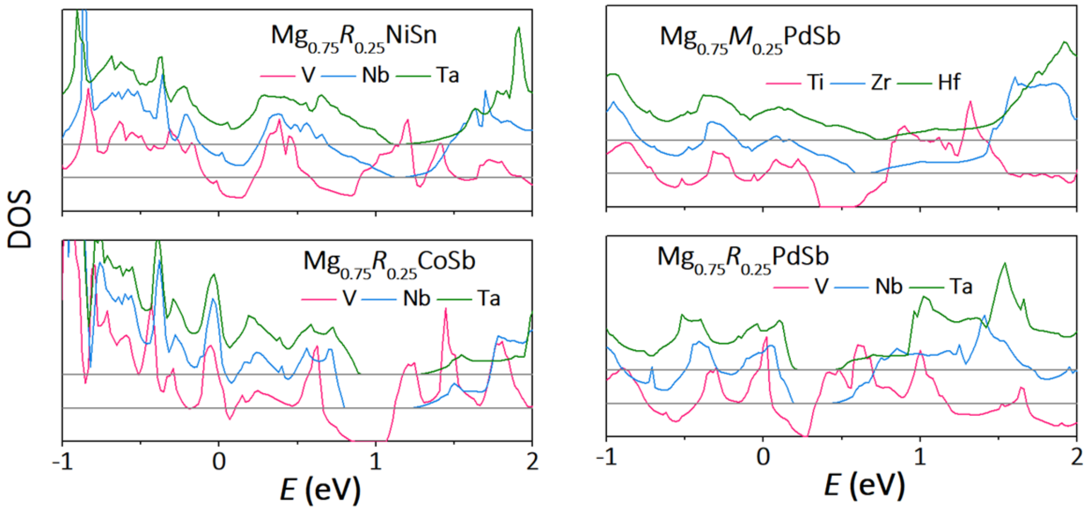

Figure S8. The density of states of $Mg_{0.75}R_{0.25}NiSn$, $Mg_{0.75}R_{0.25}CoSb$, $Mg_{0.75}R_{0.25}PdSb$ (*R* = V, Nb and Ta) and $Mg_{0.75}M_{0.25}PdSb$ (*M* = Ti, Zr and Hf).

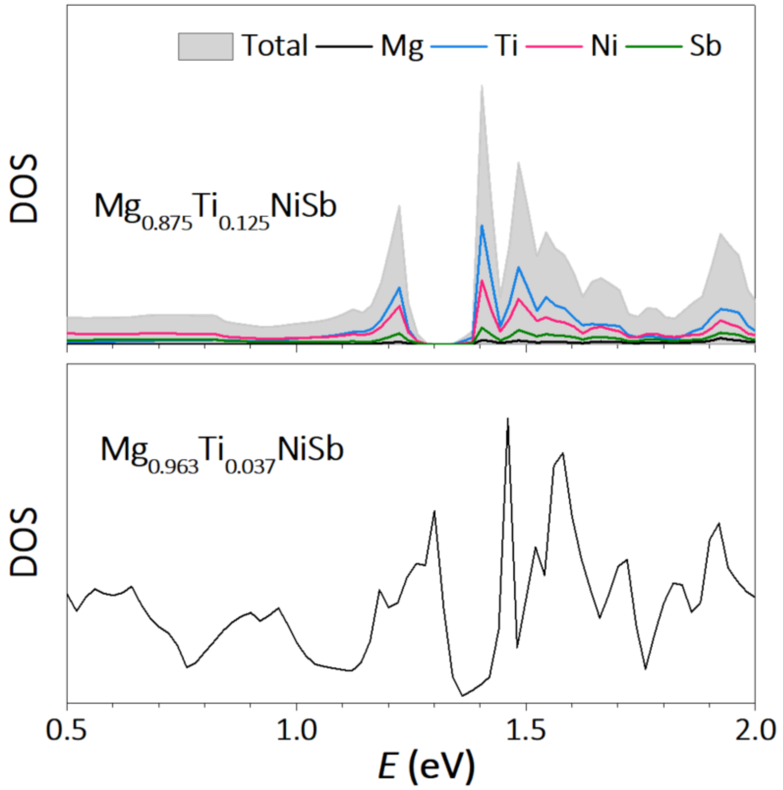

Figure S9. The density of states of $Mg_{0.875}Ti_{0.125}NiSb$ and $Mg_{0.963}Ti_{0.037}NiSb$. $Mg_{0.875}Ti_{0.125}NiSb$ has the energy bandgap, while $Mg_{0.963}Ti_{0.037}NiSb$ does not.

3. References

[1] P. Nan, A. Li, L. Cheng, K. Wu, Z. Liang, F. Lin, C. Fu, T. Zhu, B. Ge, *Mater. Today Phys.* **2021**, *21*, 100524.

[2] a) G. Kresse, J. Furthmüller, *Phys. Rev. B* **1996**, *54*, 11169-11186; b) G. Kresse, D. Joubert, *Phys. Rev. B* **1999**, *59*, 1758-1775; c) P. E. Blöchl, *Phys. Rev. B* **1994**, *50*, 17953-17979.

[3] J. P. Perdew, K. Burke, M. Ernzerhof, *Phys. Rev. Lett.* **1996**, *77*, 3865-3868.

[4] J. Heyd, G. E. Scuseria, *J. Chem. Phys.* **2004**, *121*, 1187-1192.

[5] V. Wang, N. Xu, J.-C. Liu, G. Tang, W.-T. Geng, *Comput. Phys. Commun.* **2021**, *267*, 108033.

[6] a) P. V. C. Medeiros, S. S. Tsirkin, S. Stafström, J. Björk, *Phys. Rev. B* **2015**, *91*, 041116; b) P. V. C. Medeiros, S. Stafström, J. Björk, *Phys. Rev. B* **2014**, *89*, 041407.

[7] a) M. K. Brod, S. Anand, G. J. Snyder, *Adv. Electron. Mater.* **2022**, *8*, 2101367; b) S. Öğüt, K. M. Rabe, *Phys. Rev. B* **1995**, *51*, 10443-10453.

4. Author Contributions

A. Li, C. Fu, and T. Zhu designed the project. A. Li prepared the samples and carried out the structural and transport characterizations, and did the first-principles calculations with some inputs from M. K. Brod, Y. Wang, S. Han and Z. Gao. K. Hu, P. Nan and B. Ge performed the STEM characterization. A. Li and C. Fu analyzed the data and wrote the original manuscript with discussions with M. K. Brod, S. Anand and G.J. Snyder. T. Zhu supervised the project. All the authors reviewed and edited the manuscript.
